# Assessing the effects of repeated handling on the physiology and condition of semi‐precocial nestlings

**DOI:** 10.1111/ibi.12402

**Published:** 2016-08-17

**Authors:** Hannah Watson, Mark Bolton, Britt J. Heidinger, Winnie Boner, Pat Monaghan

**Affiliations:** ^1^ Institute of Biodiversity, Animal Health and Comparative MedicineUniversity of GlasgowGlasgowG12 8QQUK; ^2^ RSPB Centre for Conservation ScienceThe Royal Society for the Protection of BirdsThe LodgeSandyBedfordshireSG19 2DLUK; ^3^ Biological Sciences DepartmentNorth Dakota State UniversityFargoND58108USA; ^4^ Present address: Evolutionary EcologyDepartment of BiologyLund UniversitySE‐22362LundSweden

**Keywords:** conservation physiology, glucocorticoids, storm‐petrel, stress, telomeres

## Abstract

Repeated exposure to elevated levels of glucocorticoids during development can have long‐term detrimental effects on survival and fitness, potentially associated with increased telomere attrition. Nestling birds are regularly handled for ecological research, yet few authors have considered the potential for handling‐induced stress to influence hormonally mediated phenotypic development or bias interpretations of subsequent focal measurements. We experimentally manipulated the handling experience of the semi‐precocial nestlings of European Storm Petrel *Hydrobates pelagicus* to simulate handling in a typical field study and examined cumulative effects on physiology and condition in late postnatal development. Neither baseline corticosterone (the primary glucocorticoid in birds), telomere length nor body condition varied with the number of handling episodes. The absence of a response could be explained if Storm Petrels did not perceive handling to be stressful or if there is dissociation of the hypothalamic–pituitary–adrenal axis from stressful stimuli in early life. Eliciting a response to a stressor may be maladaptive for cavity‐dwelling young that are unable to escape or defend themselves. Furthermore, avoiding elevated overall glucocorticoid exposure may be particularly important in a long‐lived species, in which accelerated early‐life telomere erosion could impact negatively upon longevity. We propose that the level of colony‐wide disturbance induced by investigator handling of young could be important in underlining species‐specific responses. Storm Petrel nestlings appear unresponsive to investigator handling within the limits of handling in a typical field study and handling at this level should not bias physiological and morphological measurements.

## Introduction

Wild birds are routinely handled for the purposes of recording morphological, physiological and behavioural metrics, the collection of samples for molecular analyses and the deployment of tracking devices. Acute effects of handling are reasonably well studied and include capture myopathy (Ponjoan *et al*. 2008), increased breathing rate and body temperature (Carere & van Oers 2004) and elevated glucocorticoid (GC) levels (Wingfield *et al*. 1995). Although long‐term effects, including increased energy expenditure, reduced survival and lower productivity, have been associated with the handling of birds (Gauthier‐Clerc *et al*. 2004, Sharpe *et al*. 2009, Barron *et al*. 2010, Casas *et al*. 2015), those studies were carried out in the context of tagging and were not able to separate the effects of handling from those of tagging. However, repeated handling in the absence of tagging has been shown to have long‐term effects on behavioural traits in captive birds (van Oers & Carere 2007).

Despite evidence of both acute and chronic effects of handling, few avian studies have considered the possibility for repeated capture and handling to bias subsequent interpretations of physiological or demographic data (see mammalian studies by Haydon *et al*. 1999, Clinchy *et al*. 2001). Furthermore, studies have focused on the effects of handling adult birds, yet it is well understood that early‐life experiences can have profound effects on hormonally mediated phenotypic development that can result in long‐term effects on key fitness‐related traits (Lindström 1999, Dufty *et al*. 2002, Monaghan 2008). Vertebrates respond to an acute stressor via activation of the hypothalamic–pituitary–adrenal (HPA) axis, resulting in an increased secretion of GCs (Wingfield *et al*. 1998, Wingfield & Kitaysky 2002). A short‐term increase in GC secretion can be beneficial, promoting behaviours that enhance survival (Wingfield & Kitaysky 2002, Romero 2004). However, repeated activation of the HPA axis (as a result of persistent or repeated exposure to a stressor) can lead to chronic elevation of GCs and life‐long changes in the functioning of the HPA axis, possibly via the disruption of negative feedback mechanisms (Meaney *et al*. 1988, Romero 2004). Chronic elevation of GC levels has deleterious consequences for growth, reproduction, immune function and cognitive development (Wingfield *et al*. 1998, Sapolsky *et al*. 2000, Kitaysky *et al*. 2003). In developing young, the costs of eliciting an adrenocortical response are thus more likely to outweigh the benefits.

Several studies of neonatal handling in captive and domesticated mammals and birds clearly demonstrate how early‐life experiences can modify development of the HPA axis. Regular handling during early postnatal development has been repeatedly shown to result in a reduction of baseline and/or stress‐induced GCs in both the short term as well as later adult life (Meaney *et al*. 1988, 1991, Hemsworth *et al*. 1994, Collette *et al*. 2000, Weaver *et al*. 2000, Adams *et al*. 2005). Fewer studies have considered the effects of handling in wild birds and have revealed different outcomes. In altricial young, regular handling induced a dampening of the stress response in American Kestrels *Falco sparverius* (Whitman *et al*. 2011) and Eastern Bluebirds *Sialia sialis* (Lynn *et al*. 2013), whereas European Shag *Phalacrocorax aristotelis* nestlings showed increased sensitivity of the HPA axis but no change in baseline GCs (Herborn *et al*. 2014). Neither baseline nor stress‐induced levels were affected in semi‐precocial nestlings of the Thin‐billed Prion *Pachyptila belcheri* (Quillfeldt *et al*. 2009), Black‐legged Kittiwake *Rissa tridactyla* (Brewer *et al*. 2008) or Leach's Storm Petrel *Oceanodroma leucorhoa* (Fiske *et al*. 2013).

It has been suggested that long‐term fitness costs associated with early exposure to chronic stress and elevated GCs could be mediated via accelerated telomere attrition during development (Epel *et al*. 2004, 2006), although only recently have studies started to investigate the links between GCs and telomeres (Haussmann *et al*. 2012, Herborn *et al*. 2014, Haussmann & Heidinger 2015). Telomeres comprise highly conserved non‐coding DNA sequences at the ends of eukaryotic chromosomes (Blackburn 2005). In the absence of the enzyme telomerase, telomeres shorten with each round of somatic cell division and, upon reaching a critical length, trigger cellular senescence (Blackburn 2005). The accumulation of senescent cells contributes to tissue and organ dysfunction and age‐related pathologies (Campisi 2005). Telomere length (TL) declines progressively with age in birds (Bize *et al*. 2009, Heidinger *et al*. 2012) and individuals with the shortest telomeres or the highest attrition rate have the poorest survival prospects (Haussmann *et al*. 2005, Bize *et al*. 2009, Salomons *et al*. 2009, Heidinger *et al*. 2012). Telomere loss is thought to be greatest during early life, presumably as a result of the rapid growth and cell division that occurs at this time (Zeichner *et al*. 1999, Salomons *et al*. 2009, Heidinger *et al*. 2012). Exposure to stressful or unfavourable conditions during development has been associated with accelerated telomere attrition and/or short TL in birds (Hall *et al*. 2004, Geiger *et al*. 2012, Boonekamp *et al*. 2014, Herborn *et al*. 2014, Reichert *et al*. 2014, Watson *et al*. 2015), possibly mediated by increased oxidative damage (von Zglinicki 2002, Haussmann *et al*. 2012). Environmentally induced variation in early‐life TL may therefore be particularly important in shaping individual phenotypic and life‐history development, yet little is known about how variation in the early‐life environment influences telomere loss during development.

The objective of this study was to quantify the cumulative effects of repeated handling during postnatal development on baseline corticosterone (CORT, the main GC in birds), TL and body condition in the European Storm Petrel *Hydrobates pelagicus*. We have previously shown that environmental conditions affected telomere dynamics in nestlings of this species, with unfavourable conditions resulting in accelerated attrition rates (Watson *et al*. 2015). Only one study to date has quantified telomere loss as a physiological cost associated with neonatal handling: daily handling of altricial European Shag nestlings gave rise to increased sensitivity of the HPA axis and increased telomere loss (Herborn *et al*. 2014). Whereas Shags generally nest in the open and rear altricial nestlings, Storm Petrels nest in cavities and their offspring are semi‐precocial. The presence of an investigator in a Shag colony, handling young, causes widespread disturbance of both adults and young throughout the colony, which may influence how stressful handling is for nestlings. In contrast, there is no colony‐wide disturbance caused by handling Storm Petrel nestlings. We sought to quantify the cumulative effects of handling at a level typical of a scientific study in which young may be captured a few times, for example to quantify growth or collect repeated blood samples. Many handling studies have employed a frequency of handling far exceeding that typically employed in research and monitoring. A further aim was to assess the potential for effects of handling to be threshold‐dependent, which (as far as we are aware) has only been considered in a single study of altricial nestlings (Lynn *et al*. 2013). Therefore, rather than simply comparing handled vs. unhandled, our experimental design included a range in the cumulative number of handling episodes to which young were exposed, facilitating identification of a possible threshold above which physiological responses could be induced.

## Methods

### Study site and species

The study was conducted at the island of Mousa, located in the Shetland archipelago, UK (60°00′N, 01°10′W). The Storm Petrel is a nocturnal, colonial seabird belonging to the Procellariiformes. The young are semi‐precocial (Starck & Ricklefs 1998): although nestlings hatch with a layer of down and can thermoregulate within a few days of hatching, they are dependent on their parents for food and do not leave the confines of the underground nest until fledging at 65–70 days (Davis 1957). The single chick is brooded for *c*. 7 days, following which the chick remains alone in the nest during daylight hours and parents return to provision the chick at night. Experimental handling was carried out during the day and so did not directly impede parents tending nests; therefore we did not expect any change in parental care in response to handling. Furthermore, as Storm Petrels nest out of sight in cavities, there is no colony‐wide disturbance caused by investigator handling of young. The single‐egg clutch of this species also enables the isolation of experimental effects from potentially confounding effects of the within‐nest environment (e.g. sibling competition; Love *et al*. 2003b).

### Experimental manipulation of handling

In mid‐ to late August 2011, 26 nests occupied by an unbrooded chick were identified. Nests were all located at least 150 m from areas of human recreational activity, and consequently exposed to very low levels of human disturbance. Nests had not been exposed to investigator disturbance prior to this first visit. All nestlings were handled at this first encounter to enable the estimation of age from tarsus length (M. Bolton, unpubl. data). Due to the high asynchrony in timing of breeding, nestlings spanned a range of ages at the start of the experiment (median age: 16 days; range: 3–30 days). Handling was standardized across the experiment, with each handling episode lasting 3–4 min and designed to simulate handling in a typical scientific field study (e.g. during which biometrics would be recorded). Following initial handling, nestlings were subsequently exposed to between 0 and 6 additional handling episodes over the course of the experiment until late postnatal development. The treatment period (number of days elapsed between first handling and end of experiment) therefore varied depending on the age of chicks at the beginning of the experiment. Eight nestlings were left undisturbed until the end of the experiment (handling episodes = 1), and another four were handled on one further occasion at 22 days after initial handling (handling episodes = 2). The remaining nestlings (*n* = 14) were all handled approximately every 5 days until the end of the treatment period; the cumulative number of handling episodes varied between three and seven, depending on age at the start of the treatment period.

### Collection of blood and biometrics in late postnatal development

Nestlings were exposed to their assigned handling treatment until late postnatal development (median age: 45 days; range: 41–55 days). At this point, biometrics were recorded and, for the first time, blood was collected for quantification of baseline CORT and TL, and molecular sexing. Samples were collected at least 10 days prior to fledging and thus before the likely onset of a pre‐fledging rise in baseline CORT (Quillfeldt *et al*. 2007). A minimum of 5 days elapsed between the final handling episode and the time of blood sampling. Body mass was recorded to the nearest 0.1 g using a spring scale, and wing length (maximum flattened chord) was measured to the nearest 1 mm using a stopped rule once the outermost primary feather emerged from the quill sheath (*c*. 30 days). To account for differences in timings of weighings, mass was corrected to a standardized time (18:00 h) according to the age‐related rate of proportional weight loss (Bolton 1995). Body condition was calculated according to the scaled mass index of Peig and Green (2009) using standardized mass and wing chord. Whole blood was obtained by venepuncture of the brachial vein and collected in heparinized capillary tubes under licence from the UK Home Office. All blood samples (except one, which was subsequently removed from the CORT analysis) were collected within 3 min of the onset of disturbance (determined as when the investigator approached to within 1 m of nest), so can be considered representative of baseline (Romero 2004). CORT measures were not affected by the time taken to collect the blood sample (GLM: *β* = 0.23 ± 0.16, *F*_1,23_ = 2.14, *P* = 0.157). Blood samples were stored on ice for up to 8 h prior to being separated by centrifugation. Plasma and red blood were stored at < 5 °C in the field for up to 3 days before being transferred to −20 °C for up to 3 months; following this, samples were stored at −80 °C until laboratory analyses were performed. Sex was determined following the molecular method described by Griffiths *et al*. (1998).

### Quantification of baseline plasma CORT

CORT was first extracted from plasma with 2 mL diethyl ether in a dry ice–methanol bath, evaporated under nitrogen gas and re‐suspended in 300 μL of the assay buffer. Prior to extraction, plasma was spiked with 20 μL 150 ct/min [^3^H]‐CORT to calculate recovery rate. Average recovery of samples was 81%. CORT levels were determined using Corticosterone Enzyme Immunoassay Kits (Enzo Life Sciences Inc., Farmingdale, NY, USA) and according to the manufacturer's protocol. Samples were run in duplicate at a 1 : 20 dilution and repeated samples (i.e. from the same individual) were run on the same plate. The standard curve was measured in triplicate with six standards ranging from 20 000 to 15.63 pg/mL. After adding stop solution, absorbance was measured immediately using a Thermo Scientific Multiskan EX spectrophotometer (Thermo Fisher Scientific Inc., Waltham, MA, USA) at 405 nm and corrected for 570 nm. Values were corrected for initial plasma volume and individual recovery. Mean inter‐assay and intra‐assay coefficients of variation were 13.2 and 11.5%, respectively. The detection limit of the assay was 0.46 ng/mL; this was calculated by taking two standard deviations away from the mean of the total‐binding wells. Three samples fell below the detection limit and were assigned the detection limit.

### Quantification of telomere length

TL was measured in red blood cells by quantitative PCR (qPCR) using a Stratagene Mx3005P (Agilent Technologies, Santa Clara, CA, USA) according to the methods described by Cawthon (2002) and adapted for European Storm Petrels by Watson *et al*. (2015). DNA was extracted from red blood cells using Machery‐Nagel NucleoSpin Blood kits according to the manufacturer's protocol. The method gives a relative value for TL by determining the ratio of number of telomere repeats (*T*) to that of a single‐copy (or, more precisely, non‐variable in copy number) control gene (*S*), relative to a reference sample. Although the measure will include any interstitial repeats of the telomeric sequence, good correlations have been found between TL measurements including and excluding the interstitial repeats using telomere restriction fragment (TRF) analysis (Foote *et al*. 2013) and between TRF and qPCR analyses (Criscuolo *et al*. 2009, Aviv *et al*. 2011). Furthermore, measurement of TL in Leach's Storm Petrels, using the TRF method, suggests that interstitial repeats do not occur to the extent that they would influence *T*/*S* measurements (M. Haussmann, pers. comm.).

Amplification of telomere sequences was achieved using the forward and reverse primers Tel1b (5′‐CGGTTTGTTTGGGTTTGGGTTTGGGTTTGGGTTTGGGTT‐3′) and Tel2b (5′‐GGCTTGCCTTACCCTTACCCTTACCCTTACCCTTACCCT‐3′). The non‐variable copy control gene used was ornithine decarboxylase (OCD), isolated from the European Storm Petrel (GenBank: DQ881744.1), which was amplified using the primers OCD Fwd1 (5′‐GACCTTGCCATCATTGGAGT AG‐3′) and OCD Rev1 (5′‐AAGGCATCCCTATTGTTAGGTAGA‐3′) sourced from Integrated DNA Technologies (Leuven, Belgium). The qPCR was performed using 10 ng of DNA per reaction. The concentrations of primers used were 500 nm for telomere and 70 nm for OCD reactions. Telomere qPCR reaction conditions started with 15 min at 95 °C, followed by 27 cycles of 15 s at 95 °C, 30 s annealing at 58 °C and 30 s extension at 72 °C. OCD reaction conditions started with 15 min at 95 °C, followed by 40 cycles of 30 s at 95 °C and 30 s at 60 °C. For both telomere and OCD reactions, the number of PCR cycles required for accumulation of sufficient products to exceed a threshold of fluorescent signal (*C*_t_) was determined. A standard curve, run on each plate, consisted of a serial dilution of a reference sample ranging from 40 to 2.5 ng. The *C*_t_ threshold for each reaction was determined from the reference sample. All samples, including the standard curve, were run in triplicate. Amplification efficiencies (mean ± se) were 106.8 ± 3.7 and 114.5 ± 3.9% for telomere and OCD assays, respectively. All samples fell within the bounds of the standard curve. Mean *C*_t_ values were used to calculate the *T*/*S* ratio according to Pfaffl (2001). This method accounts for variation in amplification efficiencies. Inter‐ and intra‐assay variation of *C*_t_ values were 3.6 and 1.4%, respectively, for telomere reactions, and 0.82 and 0.33%, respectively, for OCD reactions. Inter‐assay variation in *T*/*S* ratio was 10.4%.

### Statistical analyses

All analyses were performed in r 3.0.0 (R Core Team 2013). Baseline CORT and TL data were log‐transformed to achieve normality. General linear models with a normal error structure were fitted to data on (i) log‐transformed baseline CORT (*n* = 25; one sample was removed – see above), (ii) log‐transformed TL (*n* = 26) and (iii) body condition (scaled mass index; *n* = 26) to examine variation in response to the number of handling events (covariate). We considered the need to control for sex (female or male) and the covariates of age at sampling, body condition (CORT and TL models only), time of day (CORT model only), length of the treatment period, age at first handling, hatching date and the number of days left undisturbed (i.e. not handled). Although the last four covariates were all highly correlated with one another (all *r *> 0.8), adopting a forward stepwise approach to model fitting allowed us to test conservatively, one by one, for potential effects of all variables. We also considered the potential for length of treatment period and age at sampling to influence the effect of handling on all response variables by testing for the respective interactions with number of handling episodes. Starting with a null model, fixed effects were added sequentially in a forward stepwise regression to reach a minimum adequate model. The criterion for inclusion of a variable was a maximum likelihood ratio *P*‐value of ≤ 0.05. Each time a new variable was added to the model, the significance of existing variables was re‐examined. All results are presented as means ± 1 se, unless otherwise stated. Power analyses were performed using the pwr package in r (R Core Team 2013).

## Results

**Figure 1:**
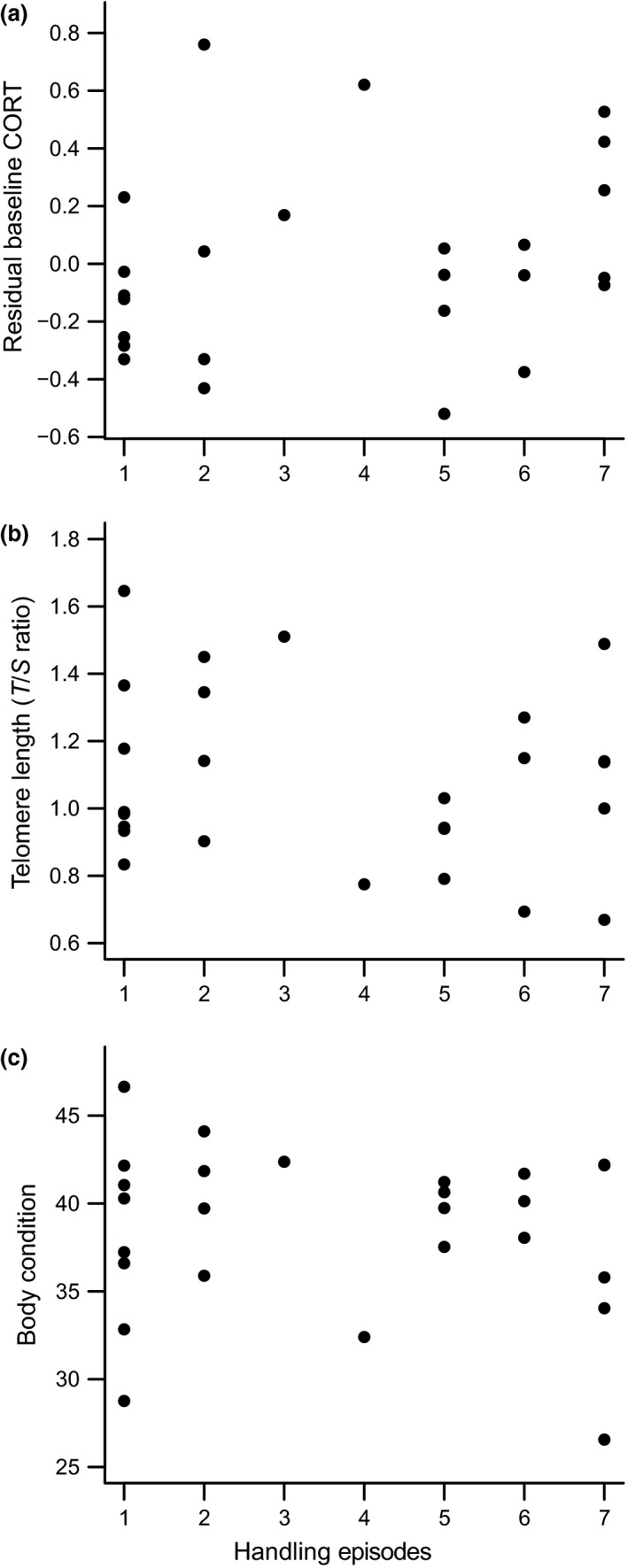
Effects of repeated handling during early life on (a) baseline plasma corticosterone (CORT;* F*_1,22_ = 1.15, *P* = 0.30, *n* = 25); (b) telomere length (*F*_1,24_ = 0.89, *P* = 0.36, *n* = 26); and (c) body condition (scaled mass index; *F*_1,24_ = 0.322, *P* = 0.58, *n* = 26) in late postnatal development (median age: 45 days). Handling is the cumulative number of handling episodes prior to the quantification of biometrics and physiological measures. Values plotted in (a) are partial residuals (calculated according to Larsen & McCleary 1972), having accounted for the effect of body condition on baseline CORT.

Neither baseline CORT (Fig. 1a; mean ± se = 1.44 ± 0.16; *β* = 0.031 ± 0.03, *F*_1,22_ = 1.15, *P* = 0.30, corrected for a negative relationship with body condition: *β* = −0.037 ± 0.01, *F*_1,23_ = 6.25, *P* = 0.02), TL (Fig. 1b; mean ± se = 1.09 ± 0.05; *β* = −0.014 ± 0.02, *F*_1,24_ = 0.89, *P* = 0.36) nor body condition (Fig. 1c; mean ± se = 38.53 ± 0.92; *β* = −0.222 ± 0.39, *F*_1,24_ = 0.322, *P* = 0.58) varied with the cumulative number of handling episodes, ranging from 1 to 7. None of the other explanatory variables explained a significant amount of the variation in either TL or body condition. There was no effect of either treatment period, age at first handling, hatching date or days not handled on baseline CORT (all *P *> 0.15), TL (all *P *> 0.41) or body condition (all *P *> 0.56). Although the length of the treatment period and age at sampling varied among individuals and with the number of handling episodes, neither variable influenced the effect of handling on either CORT (all *P* > 0.9), TL (all *P* > 0.2) or body condition (all *P* > 0.6), as tested for by the respective interactions. Power analyses revealed that, for a power of 0.8 and *α* of 0.05, the minimum detectable effect sizes were 0.44, 0.33 and 0.33, respectively, for the dependent variables of CORT, TL and body condition.

## Discussion

With the increasing use of physiological markers for assessing population status and addressing conservation issues, it is crucial that more studies consider the potential for repeated handling to influence focal measures and physiological development of neonates. Our results demonstrate that Storm Petrel nestlings were robust to handling as frequently as every 5 days and up to a maximum of seven times during postnatal development. Although an age‐related increase in baseline CORT has been demonstrated within the week prior to fledging in another procellariiform species (Quillfeldt *et al*. 2007), blood samples were collected from Storm Petrels at least 10 days prior to fledging and analyses confirmed that age did not influence our interpretation of handling effects. Power analyses suggest the sample size was sufficient to detect effects of a magnitude in the range of 33–44% with a power of 0.8; as previous studies have shown large differences (> 50%) in baseline GCs between handled and unhandled birds (Adams *et al*. 2005, Lynn *et al*. 2013), we anticipate that our study design was adequate to detect effects if they occurred. Within the limits of a typical scientific field study, investigator handling should therefore not bias physiological or morphological measurements in the focal species.

Rather than simply comparing handled vs. unhandled nestlings, our experimental design allowed for detection of threshold‐dependent effects. Within the range of handling experience applied, we found no evidence for effects of handling experience being manifest beyond a threshold of cumulative handling episodes. We are aware of only one previous study that considered the possibility for threshold effects of handling nestling birds and it was inconclusive in its findings (Lynn *et al*. 2013). Heidinger *et al*. (2006) also found no evidence for threshold effects associated with handling of adult Common Terns *Sterna hirundo*, finding no effect of recapture history on either baseline or stress‐induced CORT.

One possible explanation for the absence of any cumulative effects of repeated handling is that nestlings did not perceive handling to be stressful and did not display a short‐term elevation of GCs typical of an acute stress response. Nestlings face a trade‐off between the costs and benefits of eliciting an adrenocortical response, and strict regulation of the HPA axis during development may be necessary to avoid the potentially deleterious costs associated with increased GC exposure. Indeed, a hyporesponsive period to stress has been demonstrated in many vertebrates during early development (e.g. mammals: Sapolsky & Meaney 1986, fish: Barry *et al*. 1995, birds: Wada *et al*. 2007), including in semi‐precocial nestlings of species related to the Storm Petrel (Adams *et al*. 2008, Quillfeldt *et al*. 2009, Fiske *et al*. 2013). It is probably maladaptive for cavity‐dwelling chicks, such as those of Storm Petrels, which are unable to escape or defend themselves in the event of a predation attempt, to elicit an adrenocortical response to an acute stressor. If elicitation of an acute stress response yields high costs and low benefits, dissociation of the HPA axis from stressful stimuli in early life would be favoured, reducing a nestling's overall exposure to CORT during the vulnerable period of hormonally mediated phenotypic development. This may be particularly important in a long‐lived species such as the Storm Petrel, in which accelerated early‐life telomere erosion could impact negatively upon longevity (Bize *et al*. 2009, Heidinger *et al*. 2012).

Despite being capable of eliciting an adrenocortical response to a standardized stressor (albeit somewhat reduced compared to adults), Fiske *et al*. (2013) demonstrated that daily handling had no effect on either baseline or stress‐induced CORT levels or growth rate in nestlings of the Leach's Storm Petrel. Not only was handling at a high intensity (daily) in this study, but it was also confined to the first half of postnatal development (maximum of 29 days post‐hatch). Our study demonstrates that, in the European Storm Petrel, less frequent handling (at a level more typical of field research) extended into late postnatal development (maximum of 43 days post‐hatch) also has no effect on baseline CORT in Storm Petrels.

Even in the absence of modification of baseline CORT levels, repeated exposure to a stressor can cause an increase in stress responsiveness, resulting in an overall increase in exposure to GCs (Love *et al*. 2003a, Spencer *et al*. 2009). Increased exposure to handling and GCs, independently, was shown to accelerate telomere attrition in European Shag nestlings (Herborn *et al*. 2014). Having previously shown that Storm Petrel nestlings reared in unfavourable conditions experienced higher rates of telomere loss, we anticipated that, if handling were perceived to be stressful, TL would be negatively correlated with handling experience. Although we cannot exclude the possibility that handling may have affected stress‐induced levels, our study shows that exposure to regular handling during postnatal development did not give rise to reduced TL, suggesting that handling did not increase an individual's overall exposure to GCs. The absence of any differences in TL therefore further supports the idea that Storm Petrel nestlings did not perceive handling to be stressful and did not elicit a robust acute adrenocortical response in response to each handling episode.

The response of adults to investigator handling of young is likely to play an important role in shaping the response of young themselves to handling. In the case of European Shag nestlings, which demonstrated both increased GC levels and accelerated telomere loss in response to handling, postnatal handling of young induced anti‐predator responses in adults (Herborn *et al*. 2014). Furthermore, in Shags, responses are unlikely to be limited to the adults and young at the visited nest. Like Storm Petrels, Shags are also colonial nesters but, unlike Storm Petrels, Shags nest out in the open; thus, the presence of an investigator probably caused widespread disturbance within the colony, with potential knock‐on effects for parental care. In contrast, semi‐precocial Storm Petrel chicks were unattended by parents when handling was carried out and visiting one nest does not generate disturbance elsewhere within the colony.

Aside from chronic elevation of circulating GCs, repeated exposure to stressors can also modify GC secretion via the process of acclimation (habituation), with the consequence that individuals no longer respond in the same way to the stressor (Meaney *et al*. 1988, Romero 2004, Whitman *et al*. 2011). A dampening of the acute stress response following exposure to repeated neonatal handling has previously been attributed to acclimatization in captive and domesticated vertebrates (Meaney *et al*. 1988, Collette *et al*. 2000). As we did not measure stress‐induced CORT levels, we cannot exclude the possibility that handling may have affected the sensitivity of the HPA axis, which could permanently modify an individual's responsiveness to stress (Love *et al*. 2003a, Spencer *et al*. 2009). While permanent modification of the functioning of the HPA axis could be important for long‐term fitness, chronic elevation of baseline GCs is associated with numerous immediate and deleterious effects, the consequences of which may be particularly severe in developing young (Sapolsky *et al*. 2000). The recording of baseline measures is quicker and easier as well as more practically integrated into field studies, compared with the collection of stress‐induced measures, which involves restraining animals for up to 30 min or longer, presumably incurring greater stress to individuals. Furthermore, the optimum time for detecting the peak of the response is unknown and can vary greatly both between and within species, depending on age (Wada *et al*. 2007, Quillfeldt *et al*. 2009) or body condition (Heath & Dufty 1998).

Although the present study suggests that Storm Petrel nestlings are robust to handling within the range of handling intensity applied in a typical scientific field study, this is not consistent across all avian species. Our results suggest that nestlings are able to avoid the short‐ and long‐term deleterious effects associated with increased GC exposure and accelerated telomere attrition during early life, which may be particularly important in a long‐lived species such as the Storm Petrel. We cannot be sure what underlies the absence of a response, but the lack of a response by parents or at the colony level is likely to be important in underlining species‐specific responses of young to handling. Of course, the possibility for variation in sensitivity to handling between populations cannot be discounted and history of stress‐exposure may be important in the evolution of responses at the population level.

This study was funded by a Biotechnology and Biological Sciences Research Council studentship (BB/F016700/1) to H.W. and a European Research Council Advanced Grant (AdG 268926) to P.M. Fieldwork expenses were supported by the Louise Hiom Trust (to H.W.). We are very grateful to Tom and Cynthia Jamieson (Mousa Ferry), RSPB Shetland, Martin Heubeck, Alan Pottinger and Scottish Natural Heritage for logistical support in the field. We thank Hannah Taylor for assistance with data collection and Aileen Adam, Robert Gillespie, Kate Griffiths and Becky Watson for support in the laboratory.
